# Perverted Appetite and Impotence

**Published:** 1892-03

**Authors:** C. M. Boger

**Affiliations:** Parkesburg, W. Va.


### PERVERTED APPETITE AND IMPOTENCE.

C. M. Boger, M. D., Parkesburg, W. Va.

There are three cures in my case-book that may be worth publishing, if you think so; here they are at your disposal:

Perverted Appetite.—Mrs. D., æt. twenty-four, applied to me on several occasions for the relief of an inordinate craving for a certain brownish-red clay, which she procured from the creek bottoms and ate in large quantities. Without further thought I prescribed Alumina, with no result. Nitric-acid relieved it, but only temporarily; she now became pregnant and ceased treatment. Two months after delivery, she applied for the relief of the following symptoms, having ceased to expect any help for her strange appetite, which continued undiminished: Aching in left ovary, going to the right, worse in the morning, painful coition, swelling of entire body, slow throbbing in occiput, worse morning and evening and from emotions; *aching* in bladder, and *tenesmus* following *scant*, frequent urinations, urine throws down a creamy sediment and is covered with a *greasy* pellicle; cough, provoked by lying down at night; alternate diarrhœa and constipation, the diarrhœa is watery and gushing; burning sensation in face and dark circles under eyes. She received *Thuja*$^{200}$, followed by Sac-lac., and recovered entirely, the desire for the clay disappearing also, and it has never since returned. It is to be noted in this direction, that no such symptom seems to be present in the pathogenesis of Thuja, and that the clays eaten by those addicted to this habit are reputed to contain arsenic.

IMPOTENCY.—Mr. E., æt. thirty-eight, came from allopathic hands, where he had failed to obtain relief for the following condition: Urine dark and heavy; after urinating, spermatic fluid escapes; burning in bladder and urethra, must *wait* some time for urine to start; sleepless; emission of semen before erection is complete, and therefore no pleasure in coitus; prostatic discharge after stool. ℞. *Sepia*$^{200}$, four powders, and Sac-lac. cured him entirely, and made a convert of him.

IMPOTENCY.—Mr. C., æt. forty, also came from allopathic hands, where he obtained no relief; has dribbling of urine after urination, total extinction of sexual desire, loss of prostatic fluid during any exertion; denies all history of gonorrhœa. Received Cannabis-ind.$^{3x}$, which relieved him for two months; this was followed by a relapse. He now received Cannabis-ind. $^{10x}$, which wrought a complete cure.

